# Efficacy and association of hyperbaric oxygen therapy combined with butylphthalide and oxiracetam with serum levels of inflammatory markers in vascular cognitive impairment after acute ischemic stroke

**DOI:** 10.12669/pjms.39.3.6828

**Published:** 2023

**Authors:** Quanbin Dong, Mingxiu Wu, Wenge Hu

**Affiliations:** 1 Quanbin Dong, School of Health and Health, Dongguan Polytechnic, Dongguan 523000, Guangdong, China.; 2 Mingxiu Wu, Department of Gynecology and Obstetrics, Dongguan City People’s Hospital, Dongguan 523000, Guangdong, China.; 3 Wenge Hu, Department of Gynecology and Obstetrics, Dongguan Dongcheng People’s Hospital, Dongguan 523000, Guangdong, China

**Keywords:** Hyperbaric oxygen therapy, Butylphthalide, Oxiracetam, Post-stroke cognitive impairment

## Abstract

**Objectives:**

This study evaluated the efficacy of hyperbaric oxygen therapy (HBOT) combined with butylphthalide (NBP) and oxiracetam (OXR) for vascular cognitive impairment after acute ischemic stroke and investigated the association between such combination therapy and the serum levels of inflammatory markers.

**Methods:**

This was a prospective study which included eighty patients with post-AIS cognitive impairment (PAISCI) treated in Dongguan City People’s Hospital from January 2020 to January 2022. They were randomized into study group and control group. The control group was provided with conventional therapy consisting of NBP for intravenous transfusion and oral OXR, while the study group received combination therapy of HBOT, NBP, and OXR. A comparison was drawn between the two groups regarding clinical outcomes, levels of recovery of cognitive and neurological function and intelligence, changes in inflammatory markers, and incidence of adverse drug reactions (ADRs).

**Results:**

The response rate of the study group was significantly higher than that of the control group (p=0.04). The cognitive function scores of the study group were significantly better than those of the control group at the end of treatment (p<0.05). The post-treatment levels of inflammatory markers were significantly reduced in the study group when compared with the control group (p<0.05). At two weeks after treatment, the ADR rate of study group was significantly lower than the control group (p=0.03).

**Conclusions:**

The combination therapy of HBOT, NBP, and OXR demonstrates robust efficacy in patients with PAISCI. It is deemed to be a safe and effective treatment regimen.

## INTRODUCTION

A cerebral infarction (also known as ischemic stroke) is a common neurological disorder most frequently seen in the middle-aged and elderly populations, and vascular cognitive impairment (VCI) is one of the primary sequelae following a cerebral infarction. Central nervous system (CNS) small vessel disease is reported to cause 25% of strokes and 45% of dementia cases, with the prevalence growing with age and affecting about 5% of the age group above 50 years old to almost 100% of the elderly aged 90 years and older.^1^ Lesions, depending on location, can induce cognitive impairment, dementia, and mood disorders. Stroke survivors are prone to cognitive impairment and at high risk of dementia; however, the exact cause remains unknown.^2^ Without any specific remedy or treatment protocol, it is of great importance to prevent the occurrence of cognitive impairment or dementia and treat the affected group with combination therapy.^3^ Oxiracetam (OXR) represents an emerging kind of nootropics with claimed benefits to improve dysmnesia, repair damaged nerve cells, protect brain tissue from hypoxia, and enhance learning, while its efficacy requires corroboration.^4^

Butylphthalide (NBP) is shown to alleviate nerve system impairment and blood-brain barrier (BBB) damage by inhibiting the macrophage/microglia polarization from pro-inflammatory M1 to anti-inflammatory M2 phenotype^5^ and to improve daily living abilities and neurological function for active participation in everyday activities by increasing plasma three-mercaptopyruvate sulphurtransferase (Three-MST) and reducing 42-amino acid β amyloid (Aβ42).^6^ Therefore, NBP exhibits some therapeutic effects on severe cognitive dysfunction syndrome caused by hypoperfusion of brain tissue^7^ Hyperbaric oxygen therapy (HBOT) has been extensively used as a neuroprotective remedy to promote the recovery of damaged neurons. Results of animal experiments^8^ suggested that HBOT could protect rats with mild cognitive impairment (MCI) from early cognitive dysfunction, probably via the extracellular-regulated kinase (ERK) signaling pathway that facilitates the inhibition of cell apoptosis and cognitive function support. In the study reported herein, HBOT was combined with NBP and OXR to treat VCI after acute ischemic stroke (AIS), and the combination therapy showed considerable application value in the clinical setting.

## METHODS

In this prospective study which included eighty patients admitted to Dongguan City People’s Hospital from January 2020 to January 2022 due to post-AIS cognitive impairment (PAISCI) were included. They were randomly assigned to a study group and a control group, with each group having 40 patients. The study group consisted of 27 male and 13 female patients with an average age of (67.25 ± 3.51) years (range: 62-74 years). The control group had 25 male and 15 female patients with an average age of (66.80 ± 3.65) years (range: 60-73 years). Without significant differences in the baseline characteristics, the two groups showed a high degree of comparability (Table-I). The study was approved by the Institutional Ethics Committee of Dongguan City People’s Hospital (No.:2022015; Date: April 12, 2022), and written informed consent was obtained from all participants.

**Table-I:** Baseline characteristics between the study group and the control group (*χ̅*±*S*, n =40).

Indicator	Study Group	Control Group	t/χ^2^	P
Age (yr)	67.25±3.51	66.80±3.65	0.56	0.58
Male (n[%])	27(67.5%)	25(62.5%)	0.22	0.64
Disease course (d)	6.81±2.43	7.10±2.66	0.51	0.60
Past medical history				
Hypertension	16(40%)	17(42.5%)	0.05	0.82
Diabetes	13(32.5%)	16(40%)	0.49	0.45
Cardiac disease	7(17.5%)	4(10%)	0.95	0.33
Smoking	22(55%)	20(50%)	0.20	0.65
Insobriety	11(27.5%)	15(37.5%)	0.91	0.34
NIHSS	11.53±3.71	12.01±4.35	0.53	0.60
MMSE score	15.82±3.27	15.73±3.41	0.12	0.90
MoCA score	17.32±2.54	16.59±2.41	1.32	0.19

P >0.05.

### Inclusion criteria:

- Meeting the diagnostic and treatment criteria for AIS;^9^
- Presenting with the illness for less than 14 d;
- Moderate to severe stroke (NIH Stroke Score NIHSS≥5);^10^
- Under 75 years old;
- Presence of cognitive impairment (scoring 10 to 20 on the Mini-Mental State Exam MMSE and lower than 26 on the Montreal Cognitive Assessment MoCA);^11^
- Written consent to the study design from the patient or his/her family.

### Exclusion criteria:

- Cerebral or subarachnoid hemorrhage confirmed by CT or MRI findings;
- Comorbidities causative of cognitive changes, such as depression, Parkinson’s disease, infectious disease, brain tumors, emotional disturbance, or language disorders;
- Cerebral trauma or transient ischemic attack;
- Lack of cooperation in the tests;
- Serious cardiac, hepatic, and/or renal insufficiency lacking effective management;
- Incomplete clinical data.

Hypolipidemic, hypotensive, anticoagulant, and blood glucose-regulating agents were administered as baseline treatment for both groups. The control group was treated in a conventional fashion: 25 mg NBP, iv, bid, >50 minutes per dose (at least six hours between doses), for two weeks; 0.8 g OXR, po, bid, for 12 weeks. The study group was provided with combination therapy of HBOT, NBP, and OXR: HBOT, qd, 100 min at two ATA per cycle (compression on air for 20 minutes, oxygen breathing for 60 minutes, and decompression on air for 20 minutes), for 10 cycles as a treatment course, with an interval of three days between courses, for 12 weeks. NBP and OXR were administered in the same manner as in the control group.

Based on pre- and post-treatment analysis of MoCA score and symptoms^12^, clinical outcomes were classified as complete response (CR), partial response (PR), and no response (NR), with the response rate (RR) including both CR and PR (CR: evident improvements in clinical symptoms and vital signs, MoCA score>six; PR: improvements in clinical symptoms and vital signs to some degrees, MoCA score increased by four to six points; NR: no observable improvement in clinical symptoms or vital signs, MoCA score<four). MMSE, NIHSS, and MoCA were conducted to assess the levels of recovery of cognitive and neurological function and intelligence. On a scale from 0 to 30, MMSE scores of 27 or higher were considered normal, while scores between 21 to 26 indicated MCI, scores of 10 to 20 represented moderate cognitive impairment, and scores of nine or lower meant severe impairment. According to the 42-point NIHSS, lower scores specified a strong possibility for a good recovery of neurological function. MoCA scores of 26 or higher were deemed normal, while scores of 25 or lower (from the maximum of 30) suggested mild to severe cognitive impairment. Fasting peripheral venous blood (5 mL) was drawn from each patient before and after treatment to investigate changes in such inflammatory markers as tumor necrosis factor α (TNF-α), c-reactive protein (CRP), and interleukin-six (IL-6) using ELISA. ADR rates at two weeks after treatment were compared between the two groups.

Statistical analysis was performed using SPSS 20.0, with the measurement data being represented by (*χ̅*±*s*). Intergroup comparisons were examined with the independent-samples t-test, while intragroup comparisons were analyzed with the paired t-test. The comparison of percentages was verified using the chi-squared (χ^2^) test. P<0.05 indicated a difference of statistical significance.

## RESULTS

The results of the intergroup comparison of clinical efficacy showed that the study group had an RR of 85%, significantly higher than the RR (65%) in the control group see (p=0.04) (Table-II).

**Table-II:** Intergroup comparison of clinical efficacy (*χ̅*±*S*, n =40).

Group	CR	PR	NR	RR
Study group	18	7	9	34(85%)
Control group	13	6	7	26(65%)
χ^2^				4.27
P				0.04

P <0.05.

Before treatment, the two groups did not differ greatly in MMSE, NIHSS, and MoCA scores (p>0.05, respectively). After treatment, the study group scored higher on the MMSE and MoCA and lower on the NIHSS than the control group, with the differences between the two groups reaching a significant level (MMSE: P =0.00; MoCA: P =0.01; NIHSS: P =0.00) (Table-III).

**Table-III:** Intergroup comparison of pre- and post-treatment cognitive and neurological function and levels of intelligence (*χ̅*±*S*, n =40).

Outcome measures		Study Group	Control Group	t	P
MMSE score	Pre-treatment	15.82±3.27	15.73±3.41	0.12	0.90
	Post-treatment*	24.66±5.73	18.63±4.08	5.42	0.00
NIHSS	Pre-treatment	11.53±3.71	12.01±4.35	0.53	0.60
	Post-treatment*	8.03±2.14	10.47±3.80	3.54	0.00
MoCA score	Pre-treatment	17.32±2.54	16.59±2.41	1.32	0.19
	Post-treatment*	25.78±4.05	22.96±5.41	2.64	0.01

* P <0.05.

Differences between the two groups in the pre-treatment levels of TNF-α, CRP, and IL-6 lacked statistical significance all (p>0.05). After treatment, the TNF-α, CRP, and IL-6 levels in the study group were significantly decreased as compared with the control group, and the differences were statistically significant (p=0.00, respectively) (Table-IV). At two weeks after treatment, the ADR rate was 12.5% in the study group and 32.5% in the control group, indicating a statistically significant difference between the two groups (p=0.03) (Table-V).

**Table-IV:** Intergroup comparison of pre- and post-treatment levels of inflammatory markers (*χ̅*±*S*, n =40).

Indicator		Study Group	Control Group	t	P
TNF-ɑ (ng/L)	Pre-treatment	38.53±7.14	37.51±7.06	0.64	0.52
	Post-treatment*	9.63±3.46	13.42±5.73	3.58	0.00
CRP (mg/L)	Pre-treatment	28.73±6.48	27.94±7.07	0.52	0.60
	Post-treatment*	11.74±3.68	15.68±4.32	4.39	0.00
IL-6 (ng/L)	Pre-treatment	10.71±2.46	10.87±1.69	0.34	0.73
	Post-treatment*	4.51±1.63	7.24±2.35	6.04	0.00

* P <0.05.

**Table-V:** Intergroup comparison of ADR rates (*χ̅*±*S*, n =40).

Group	Fever	Gastrointestinal Response	Allergic Reactions	Rash	Decreased Granulocytes	Incidence (n[%])
Study group	3	0	0	1	1	5(12.5%)
Control group	2	5	3	2	1	13(32.5%)
χ^2^						4.59
P						0.03

P >0.05.

## DISCUSSION

Post-stroke vascular cognitive impairment (PSVCI) often occurs within six months from the onset of a stroke, which produces a strong impact on the patient’s quality of life and daily life activities.^13^ Weaver et al.^14^ Reported that cognitive impairment was observed in about 50% of the cases in the first year after stroke. In addition to infarct location as a potential determinant, cognitive impairment is also associated with age, blood pressure, and blood glucose.^15^ The incidence of post-stroke cognitive impairment (PSCI) has been climbing over the past few years. Evidence shows that the mortality rate is substantially higher in stroke patients with cognitive impairment than those without.^16^ The pathogenesis appears to involve: Structural damage to the brain tissue; Neurotransmitter systems with abnormal changes in acetylcholine (ACh), arterenol, dopamine, enteramine, gamma-aminobutyric acid (GABA), glutamate, and brain-derived neurotrophic factor; Inflammatory responsefree radical reactions.^17^

PSCI is defined as a multifactorial disorder that may benefit from combination therapy.^18^ ACh is one of the neurotransmitters most strongly associated with learning and memory function, and its level in the brain tissue is positively correlated with the level of intelligence. OXR, a cyclic derivative of GABA, can effectively ameliorate cognitive impairment induced by ischemic and hypoxic brain injuries and directly act on the damaged brain tissue by crossing the BBB.^19^ NBP can benefit patients with symptoms of acute anterior circulation infarction and contribute to a good prognosis. NBP treats ischemic cerebrovascular disease by modulating multiple targets involved in varied pathophysiological processes to deliver antioxidant, anti-inflammatory, anti-apoptotic, antithrombotic, and mitochondrial protective benefits.^20^

Combination of pharmacological and non-pharmacological interventions, such as individualized nursing care, psychotherapy, and HBOT,^21^ is associated with a better prognosis and improvements in PSCI symptoms. HBOT is a well-established treatment conducive to the recovery of cognitive function.^22^ Evidence shows that HBOT can enhance neural plasticity and cognitive function in stroke patients. A clinical trial^23^ pointed out that HBOT could promote cognitive improvement in dementia patients^24^ and to some extent benefit patients with vascular dementia. In this study, the combination therapy with HBOT yielded an RR of 85%, significantly higher than the RR (65%) in the control group (p=0.04). Besides, the study group scored higher on the MMSE and MoCA and lower on the NIHSS than the control group, and the differences were statistically significant (MMSE: p=0.00; MoCA: p=0.01; NIHSS: p=0.00). This indicated the positive role of HBOT in treating ischemic stroke to improve the patient’s mental state, cognitive function, level of intelligence, and daily living abilities, conforming to the results provided in other related studies.

Inflammatory responses are deemed to be a key player in the progression of PSCI.^25^ Cerebral ischemia can activate endothelial cells and accelerate the release of IL-6 and TNF-α.^26^ Kamnaksh et al.^27^ showed that VCI worsened with the increase in IL-6 and TNF-α expression in cerebrospinal fluid; a higher plasma level of IL-6 indicated smaller hippocampal gray matter volume, a marker of severity in VCI patients. In other words, inflammatory responses probably affect the hippocampal gray matter via the IL-6 pathway. TNF-α plays a crucial role in the induction of inflammatory responses, mediation of apoptosis pathways, stimulation of inflammatory cascades, and massive secretion of other cytokines such as IL-8 and IL-6, resulting in a vicious circle where these cytokines in turn aggravate tissue damage and increase the volume of an infarct. Gottfried et al.^28^ clarified that HBOT affected cellular processes, transcription factors, mitochondrial function, oxidative stress, and inflammation to varying degrees. Therefore, HBOT is considered an emerging pivotal player in 21st-century research and clinical treatment.

Laboratory animal experiments^29^ showed that HBOT increased cerebral blood flow and reduced cerebral hypoxia through enlargement of the arteriolar luminal diameter. In addition, HBOT lightened the amyloid burden by attenuating pre-existing plaques and inhibiting the formation of new plaques, which demonstrated an unequivocal correlation with cognitive improvements in the mice. A recent study^30^ viewed that HBOT prevented neuron injury through attenuation of autophagy, inflammation, and calcium overload.

These findings offer novel mechanisms of action and laboratory evidence for the clinical treatment of cognitive abnormalities. Gonzales-Portillo et al.^31^ Believed that HBOT was effective and feasible in the prevention, alleviation, and improvement of cognitive impairment, with a highly complex mechanism of action that involves endogenous antioxidant activities, and anti-inflammatory defense systems. Moreover, HBOT was shown to reduce ADRs triggered by chemicals. In this study, HBOT was employed as part of the combination therapy, and the recipients experienced substantial reductions in the inflammatory markers including TNF-α, CRP, and IL-6, and the ADR rate as compared with the control group, with the differences indicative of statistical significance (p=0.00, and p=0.03). All this reveals the clinical value of HBOT as a treatment for ischemic stroke which can modulate inflammatory responses, reduce IL-6 and TNF-α expression, and protect against ADRs.

### Limitations of the study:

It includes the lack of follow-up data which can be overcome by adding patients and follow-up data in future studies to further evaluate the treatment method in a comprehensive and objective way and provide more treatment options for patients.

## CONCLUSIONS

In conclusion, the combination therapy of HBOT, NBP, and OXR demonstrates robust efficacy in patients with PAISCI. It is deemed to be a safe and effective treatment regimen that can promote the recovery of cognitive and neurological function as well as the level of intelligence and markedly reduce ADRs and inflammatory markers.

### Authors’ Contributions:

**QD:** Designed this study, prepared this manuscript, are responsible and accountable for the accuracy and integrity of the work

**MW:** Collected and analyzed clinical data

**WH**: Data analysis, significantly revised this manuscript.
